# Persistent obstacles for return to work after COVID-19 infection – an explorative follow-up study in Sweden

**DOI:** 10.3389/fresc.2025.1628490

**Published:** 2025-09-18

**Authors:** Hilda Öhlén, Iolanda Santos Tavares Silva, Marie Gustafsson, Sara Jarl, Ann Björkdahl

**Affiliations:** ^1^ Sahlgrenska University Hospital, Goteborg, Sweden; ^2^ University of Gothenburg, Gothenburg, Sweden

**Keywords:** activities, obstacles, pandemic wave, post-COVID, profession, return to work (RTW)

## Abstract

**Design, aim and method:**

To explore in patients with postcovid-19 condition the influence of various factors on return to work in the year following diagnosis. The study had an explorative quantitative and qualitative design based on interviews with 41 hospitalized (HC) and 63 primary care (PC) COVID-19 patients. RTW was described at 3 and 12 months, and differences between groups, changes over time and possible factors explaining RTW were analysed statistically. Qualitative analyses with content analysis of interviews were performed to describe obstacles to work.

**Result:**

The obstacles for RTW were persistent symptoms such as fatigue, cognitive dysfunction and breathlessness with the consequences for work as lack of energy, decreased physical capability, decreased mental stamina, reduced cognitive ability, increased sensitivity to stress and general reduced capacity. At 12 months, 50% and 70% of patients in the PC and HC groups, respectively, had returned to full-time work, while 20% of patients in both groups had not RTW at all. To function at work, many patients expressed that they required adaptations. RTW was not dependent on the initial severity of COVID-19 or type of work. The likelihood of RTW was higher in males with COVID-19 onset during the second wave.

**Conclusion:**

RTW may be limited after COVID-19 infection, regardless of its initial severity. Women with persistent covid had greater difficulty than men in returning to work. Limitations were due to a general reduced capacity mainly caused by physical and mental fatigue, and cognitive dysfunction. However, the support from employers and the environment also has an impact on the RTW. If necessary, appropriate rehabilitation measures should be offered and adaptations of work content and the organization of the work at the workplace are of great importance.

## Introduction

COVID-19 can affect multiple organs in the body with far reaching consequences. Both patients who required hospitalisation, including those admitted to the intensive care unit (ICU), and primary care (PC) patients with a milder COVID-19 infection may experience significant residual disabilities for a long period of time (1, 2). The World Health Organisation (WHO) has defined a condition with long-lasting problems as “post-COVID-19 condition” (PCC), which “occurs in individuals with a history of probable or confirmed SARS-CoV-2 infection, usually 3 months from the onset, with symptoms that last for at least 2 months and cannot be explained by an alternative diagnosis” (3, 4). A study based on data from national registers and primary health care databases for all adult inhabitants of the two largest regions in Sweden, comprising around 40% of the Swedish population (4.1 million persons), found that 2.0% of all registered patients with COVID-19 had been diagnosed with PCC (5).

All viruses, including SARS-CoV-2, change over time and the WHO established a group in 2020, with a specific focus on SARS-CoV-2 variants, their phenotype and their impact on countermeasures in order to prioritize global monitoring and research, and to inform and adjust the COVID-19 response. They also started to characterize some virus variants as variants of interest (VOIs) and variants of concern (VOCs) to be able to inform about an increased risk to global public health (6). In Sweden, a combination of virus variants predominated from February 2020 to January 2021 (first and second waves), followed by the Alpha variant of concern (VOC) from February to June 2021 (third wave), and the Delta VOC from July to December 2021 (fourth and fifth waves (5). A Swedish study tried to describe potential virus variant-specific characteristics. They found that the cumulative incidence of PCC was lowest in the Delta VOC period but with the highest incidence rate in the same period. There were fewer diagnosed PCC cases in the Delta VOC than the other two periods (5).

A meta-analysis of the 1-year prevalence of specific post-COVID symptoms showed that the most prevalent symptoms were fatigue/weakness (28%), dyspnea (18%), arthromyalgia (26%), depression (23%), anxiety (22%), memory loss (19%), concentration difficulties (18%) and insomnia (12%) (7). Recovery was slow, with limited changes in fatigue and cognitive function from 3 months to 1 year in both hospitalised and non-hospitalised COVID-19 patients (8). Many symptoms persisted for 2 years after infection, the most common being cognitive dysfunction, sensorimotor difficulties and fatigue (9). Long-term persistent symptoms are more prevalent in females than males and in older age (10–12) and in individuals with a higher level of education (5). The acute severity of the infection has also been found to be a risk for PCC (11–13). However, the majority of the PCC cases in a study from Sweden had not been hospitalised for COVID-19 (5). PCC is not restricted to the elderly which Daugherty et al. showed in 2021 with an increased risk of developing new clinical sequelae after the acute phase of COVID-19 infection, including specific types of sequelae that are less common in other viral diseases. The greatest risk was for older adults with pre-existing conditions who were hospitalized, but younger adults (<50) without pre-existing conditions and those not hospitalized were alsoat increased risk of developing new clinical sequelae (14).

Evaluation of return to work (RTW) after COVID-19 infection also found that symptoms such as fatigue, cognitive dysfunction, shortness of breath and autonomic dysregulation were major obstacles to RTW (15, 16). In addition, women reported a larger reduction in work ability than men (16). A 2-year follow-up of patients hospitalised with COVID-19 found that over 50% of those working before COVID-19 had returned to part or full-time work, although their occupational status was significantly worse after than before COVID-19 infection (9). Moreover, around 50% of these individuals who were on sick leave at 4 months were still on sick leave at 2 years (9). The workability obstacles have been described as being multi-level, comprising fatigue, an interaction between symptoms and job, lack of control over time and content of work tasks, inappropriate sickness absence management policies, and lack of COVID-aware organizational cultures (17). Post-COVID condition has been associated with high functional impairment, particularly in social leisure activities and ability to work, which has a negative effect on their health-related quality of life (18). The present study explored RTW during the first year after onset of COVID-19 infection and tried to describe the obstacles for work activities. In Sweden the employerś responsibility for work rehabilitation is far-reaching (19), why it is important to contribute knowledge about barriers to return to work to increase understanding of how to facilitate return to work.

### Aim

The aim of the study was to explore return to work and eventual obstacles for the return in the first year after contracting COVID-19 infection, in patients with a severe onset in need of hospital care and patients with a milder onset not in need of hospitalization but seeking primary care rehabilitation due to persistent dysfunction.

Research questions:

1. To what extent do the patients in the study RTW fully or partially in the first year after contracting COVID-19?
2. Are there differences in the RTW according to sociodemographic factors such as age, sex, or type of work?
3. Which obstacles for RTW are expressed in the interviews of the patients in the study?
4. How large are the proportions of patients that the evaluators rate as having moderate or great obstacles for RTW at one year post onset of COVID-19 infection?
5. Is there a difference in RTW between those admitted to hospital compared to those with a milder onset seeking rehabilitation in the primary care?

## Materials and methods

This study was part of a larger prospective 1-year follow-up study with quantitative and qualitative data from interviews using a convergent parallel mixed-method design where quantitative and qualitative data collection occurred in parallel, and analysis started after data collection. Mixed-method is suitable to use to explore complex health processes and health care systems, exploring why and how a phenomenon occurs (20). In the present study, mixed-method was used to provide a better understanding of what hinders the return to work by complementing quantitative with qualitative data. Data was collected on hospitalised and non-hospitalised patients in Sweden who were diagnosed with COVID-19 infection during the first, second and third waves of the pandemic (7). (Reported in: FOU I Sverige Dnr 274943, 277346, https://www.researchweb.org/is/sverige/project/274943 and LECOG-COV-19/277346).

The study was approved by the Swedish Ethical Review Authority (Dnr: 2020-03222, 2021-03824) and complied with the Declaration of Helsinki. A written consent was obtained from all participants.

### Sample

Patients for the main study were adults recruited if they had been diagnosed with COVID-19 and were either admitted to hospital or underwent rehabilitation in a primary care facility in Gothenburg the second largest city in Sweden (>600,000 inhabitants). Hospitalised patients (HC, *n* = 122) were recruited from 1 July 2020 to 28 February 2021, and primary care patients (PC, *n* = 90) were recruited from 1 September 2020 to 31 August 2021. Patients were included if (a) they were able to understand and participate in cognitive screening as well as an interview on occupational performance, (b) lived independently prior to infection. Patients in the HC group were also required to have a hospital stay ≥5 days in a ward other than the ICU. Patients in the PC group were also required to have sought rehabilitation in a primary care service because of consequences following COVID-19 infection. During data collection, a third group was identified, consisting of 13 patients in the PC group with prior hospitalisation, but outside the inclusion period for HC from 1 July 2020 to 28 February 2021 (PC+ group).

**Figure 1:**
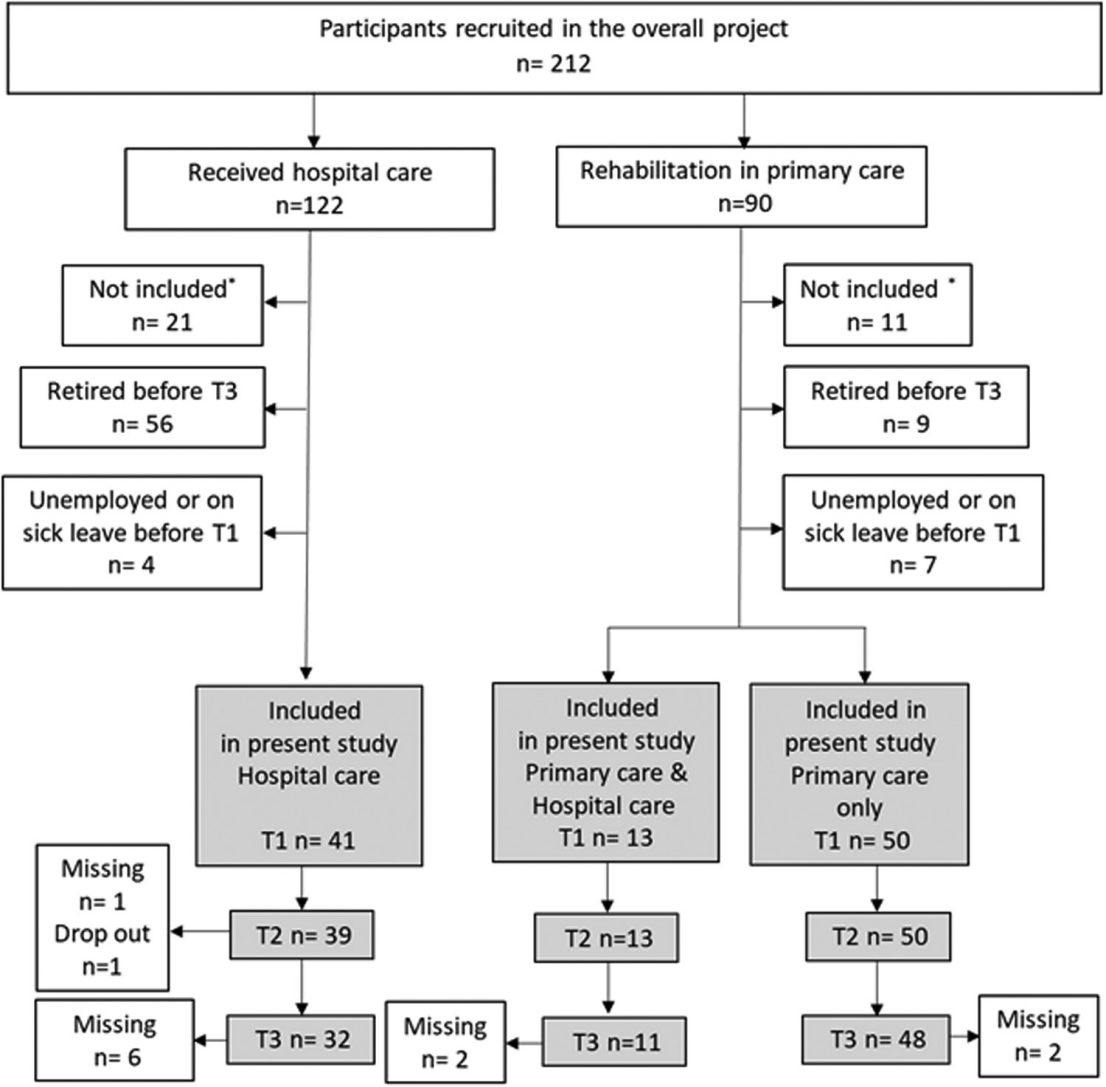
Flowchart of the participants included in this study. The grey areas indicate the flow chart of the present study.

For the present study of RTW, patients older than working age or unemployed before the onset of COVID-19 were excluded. Patients were also excluded from the analysis if (a) employment had ceased prior to follow-up or (b) data at 3- or 12-months were missing. After application of these inclusion and exclusion criteria, 104 (48.1%) of the 212 participants recruited for the main study were deemed eligible and included in the present study. These included 41 participants in the HC group, 50 in the PC group and 13 in the PC+ group. There was some change over time in the number of participants in each group assessed at 3 and 12 months. One subject in the HC group dropped out because of unemployment prior to 3 months; and one who was followed-up at 12 months, was not followed-up at 3 months, with the latter not included in comparisons at 3 and 12 months. In addition, seven subjects in the HC group could not be reached at 12 months. Two subjects each in the PC and PC+ groups dropped out prior to 12 months. Thus, at 12 months, 32 participants remained in the HC group, 48 in the PC group and 11 in the PC+ group (Figure 1). Six participants were studying at the time of admission, age m = 32,50 (SD 10,37). This included higher education, further education or studies for retraining. These participants were included as we equated studying in this way with being able to return to work.

### Data collection

#### Main study

Data were collected at baseline and 3- and 12-months after the onset of COVID-19. All patients were administered cognitive tests and were interviewed regarding symptoms of breathing, fatigue and cognitive dysfunction, and their impact on daily life activities. Four occupational therapists (OTs) were involved in the data collection and were calibrated before regarding the implementation. The same interview guide was used (Supplementary Material). Questions on the presence or absence of symptoms were answered yes or no (for quantitative analyse), with additional questions on the impact of the symptoms on the ability to perform daily life activities, further expanded with the patient's own words (for qualitative analyse). Each interview also included semi-structured questions regarding the ability to perform activities in four areas of daily life: personal care [personal activities of daily life (PADL)], household activities [instrumental activities of daily life (IADL)], leisure activities and work or study activities. The material from the interviews were used for a qualitative analyse of the patientś experiences of activity restrictions because of consequences of the disease. Based on these descriptions, the interviewers also estimated the extent to which the patient was hindered in the different activities by the consequences of COVID-19 infection, with rating scored as 0 (not at all), 1 (to some extent), or 2 (to a great extent). Analyses regarding cognition, fatigue and daily activities and the change during the first year are presented in an earlier article (8). A summary of these findings is presented below under the heading “Previously analyzed data from the same data collection in the main study”.

#### Data regarding work in the present study

In the present study interview data regarding persistent presence of symptoms (cognitive, fatigue) were used together with data regarding their occupation and obstacles for work. As we asked about occupation we also got some participants that were students in higher education which we equalled with work as it was their occupation and also where they got their income. Specific questions were posed regarding the amount of work they performed at the time of interview (0%, 25%, 50%, 75% or 100%), the type of work, obstacles to working and eventual adaptations to enable working. The participants were asked to describe whether they could perform work in the same way as before COVID-19 infection and to elaborate on these answers (8).

### Previously analyzed data from the same data collection in the main study

Data from the main study has earlier been published presenting the impact of cognitive dysfunction, fatigue, and shortness of breath, on activities of daily life after COVID-19 infection, until one year follow-up (8). These results may be of interest for the present study on RTW as it is partly the same sample why it is shortly presented here. The result showed that the levels of fatigue and cognitive dysfunction in both the HC and PC groups were high and persisted for 12 months. A significant impact on activities of daily life was also observed, with marginal changes from 3- to 12-months. Subjects in the HC group performed worse than those in the PC group in the cognitive tests, although subjects in the PC group perceived higher levels of fatigue and cognitive dysfunction. Limitations in activity, however, were greater in the PC than in the HC group (8).

### Analysis in the present study

Primary outcomes of the present study were level of RTW, factors influencing RTW, degree of working limitation and eventual differences in RTW between groups as quantitative analyses. Statistical analyses were performed using the statistical package IBM SPSS Version: 28.0.1.1. Experiences of work limitations expressed in the interviews were used for the qualitative analyse. An open-ended question in the 12-month interview was: “If any, which obstacles to resume work after the COVID infection do you still experience?”. A qualitative content analysis of the written answers to the question was conducted.

#### Descriptive analyses

Demographic factors analysed in the three groups (i.e., the HC, PC and PC+ groups) included age, sex, level of education, occupation (work or education), date of COVID-19 onset, pandemic wave, days hospitalised in the HC group, and weeks to first assessment in the PC group. Occupations were divided into three domains: white collar work (office and theoretical work), blue collar work (carpenters, practical work etc), and work in the service sector (bus driver, shop assistant). Level of education was based on those existing in the Swedish school system; 9-year compulsory school, 3-year upper secondary school or higher education such as university education. For the present study regarding the RTW we included participants that had been in higher education, which we equated with being able to return to work. The dates for different pandemic waves were in Sweden, a combination of virus variants predominated from February 2020 to January 2021 (first and second waves), followed by the Alpha variant of concern (VOC) from February to June 2021 (third wave), and the Delta VOC from July to December 2021 (fourth and fifth waves) (5).

Occupational therapists (OTs), that interviewed the participants, rated the extent to which different activity areas were restricted and for this study the ratings were based on the participants experiences of persistent symptoms and obstacles for RTW. The result of the ratings of the degree of restrictions for work are presented as proportions in each of the three groups.

#### Comparisons and regressions

Due to the low number in the *P* + group, this group was not used for comparisons. Differences in the percentages of participants in the HC and PC groups who returned to work partially or fully were compared using χ^2^ test and the Mann–Whitney U test. Within-group comparisons at 3 months and 12 months were determined using Wilcoxon signed rank tests. The probability of occurrence of an event was analyzed using the Odds ratio (OR).

Logistic regression analyses were performed to assess factors, such as age, sex and type of work, associated with RTW at one year, as well as the relationship between pandemic wave at onset of COVID-19 and RTW at one year. For the logistic regression RTW was dichotomised as 0%–25% work, considered not working, and >25%–100% work, considered as working part- or full-time. Two logistic regression models were designed because of the relatively small sample.

#### Qualitative content analysis

The open-ended questions in the interview regarding obstacles for work have been qualitative analysed with content analysis according to Granheim and Lundman (21). Meaning units describing obstacles to work were retrieved from the answers. Thereafter meaning units related to each other were given codes, which were analysed further to create categories. The categories describe what were expressed as obstacles to work. The categories include respectively subcategories that describe how these impairments and conditions manifest themselves as barriers in the work situation.

The qualitative and quantitative findings; statistical analysis and analysis of answers to open-ended question in interviews were performed separately. The integration of qualitative and quantitative data took place in the presentation of the results, according to mixed method design in the reporting level.

## Results

### Patient demographic and clinical characteristics

Participants who were hospitalised had a mean hospital stay of 37 days (±40) and were included in the study at the end of the hospital stay. Participants in the PC group were included when they contacted the local primary care rehabilitation clinic because of remaining rehabilitation needs, resulting in a variation in time from onset to first assessment (Table 1).

**Table 1:** Demographic and clinical characteristics of subjects enrolled in this study.

Participant characteristics				
Variables	In total	Hospital care	Primary care only	Hospital & primary care *n* = 13
	*n* = 104	*n* = 41	*n* = 50	
Men/Women number = *n* (%)	56/48 (54/46)	32/9 (78/22)	16/34 (32/68)	8/5 (62/38)
Age, yr mean (SD)	49.7 (10.9)	53.3 (9.61)	46.2 (11.5)	51.8 (8.5)
Education *n* (%)				
Elementary school/High school	26 (25)	15 (36.6)	8 (16)	3 (23)
After high school/Practical education	32 (31)	11 (27)	15 (30)	6 (46)
University/college	46 (44)	15 (36.6)	27 (54)	4 (31)
Occupation before COVID-19 *n* (%)	(*n* = 104)	(*n* = 41)	(*n* = 50)	(*n* = 13)
White collar work	62 (59,6)	23 (56.1)	31 (62.0)	8 (61,5)
Blue collar work	27 (26,0)	13 (31,7)	10 (20.0)	4 (30.5)
Service sector work	9 (8.7)	5 (12.2)	3 (6.0)	1 (7.7)
Student	6 (5,8)	0 (0,0)	6 (12.0)	0 (0.0)
Pandemic wave^a^ *n* (%)				
I (March-September 2020)	49 (47)	13 (32)	28 (56)	8 (62)
II (October 2020- January 2021)	45 (43)	28 (68)	14 (28)	3 (23)
III (February 2021- June 2021)	10 (10)	0 (0)	8 (16)	2 (15)
Days hospitalised mean (SD)		37 (40)		18 (11)
Time to first assessment (weeks)				
Mean (range)			16.5 (2–56)	17.0 (4–30)

^a^ Virus variants: in Sweden, a combination of virus variants predominated from February 2020 to January 2021 (first and second waves), followed by the Alpha variant of concern (VOC) from February to June 2021 (third wave), and the Delta VOC from July to December 2021 (fourth and fifth waves) (5).

About two-thirds of the subjects in the HC group were men, whereas about two-thirds of the subjects in the PC group were women (*ꭕ*^2^ = 15,95 (*p* < 0.001). Subjects in the PC group were significantly younger (*p* = 0.002, 95% CI −11.48 to −2.63) and had a higher level of education (χ^2^ = 13,47, *p* = 0,004) than those in the HC group. The PC+ group was similar to the HC group. The six students were only present in the PC group, but otherwise the distribution of different types of occupation was similar in these groups, with theoretical work being most common in all groups. About two-thirds of the subjects in the HC group fell ill during the second wave of the pandemic, but none in the third wave, whereas most subjects in the PC group fell ill during wave one, with the percentages who fell ill during waves two and three being similar. The onset of most of the patients in PC+ were in wave one (Table 1).

### Between differences of RTW in HC and PC

Comparisons of subjects working (>25%–100%) and not working (0%–25%) showed no statistically significant differences between the HC and PC groups at 3 and 12 months. Similarly, no significant between-group differences at 3 and 12 months were observed when percentage working was set at 0%, 25%, 50%, 75% or 100%.

### Within group changes of RTW from 3 to 12 months

**Figure 2:**
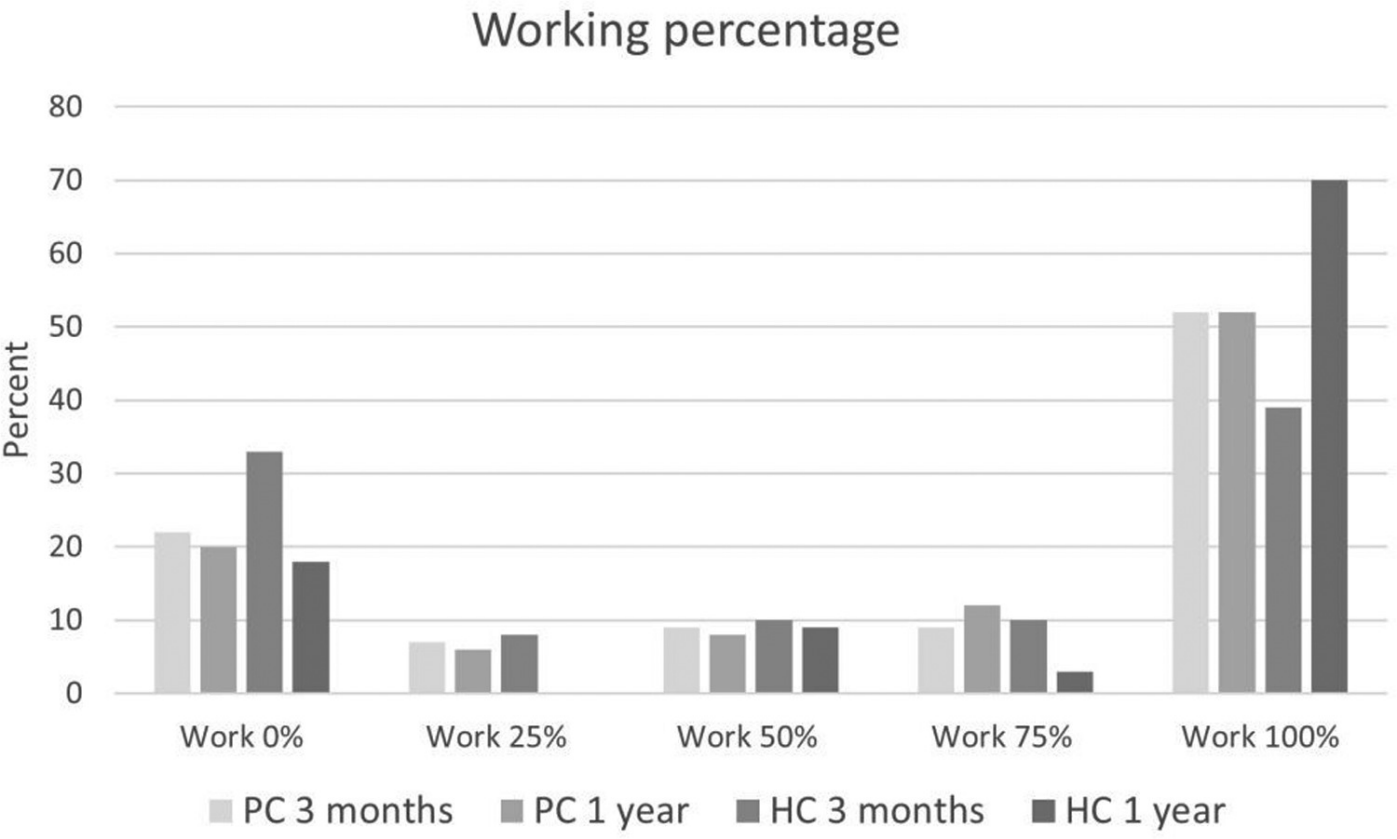
Employment rates of subjects in the HC and PC groups at 3 months and 1 year.

The changes within groups, between 3 and 12 months, in the percentage working, increased significantly in the HC (*p* = 0.01, *Z* = −2.57), but not in the PC group (*p* = 0.94, *Z* = −0.075) (Figure 2). In the PC group the percentage back in full-time work was the same at the two time points, 3 and 12 months. While, in the HC group, the percentage back in full-time work changed significantly (*Z* −2.52, *p* = 0.012) between 3 and 12 months, increasing from 38% at 3 months to 70% at 12 months. About 20% of the subjects in both the PC and HC groups were unable to RTW at 12 months. RTW in the PC+ group did not differ significantly at 3 and 12 months (Figure 2).

### Aspects of RTW

**Figure 3:**
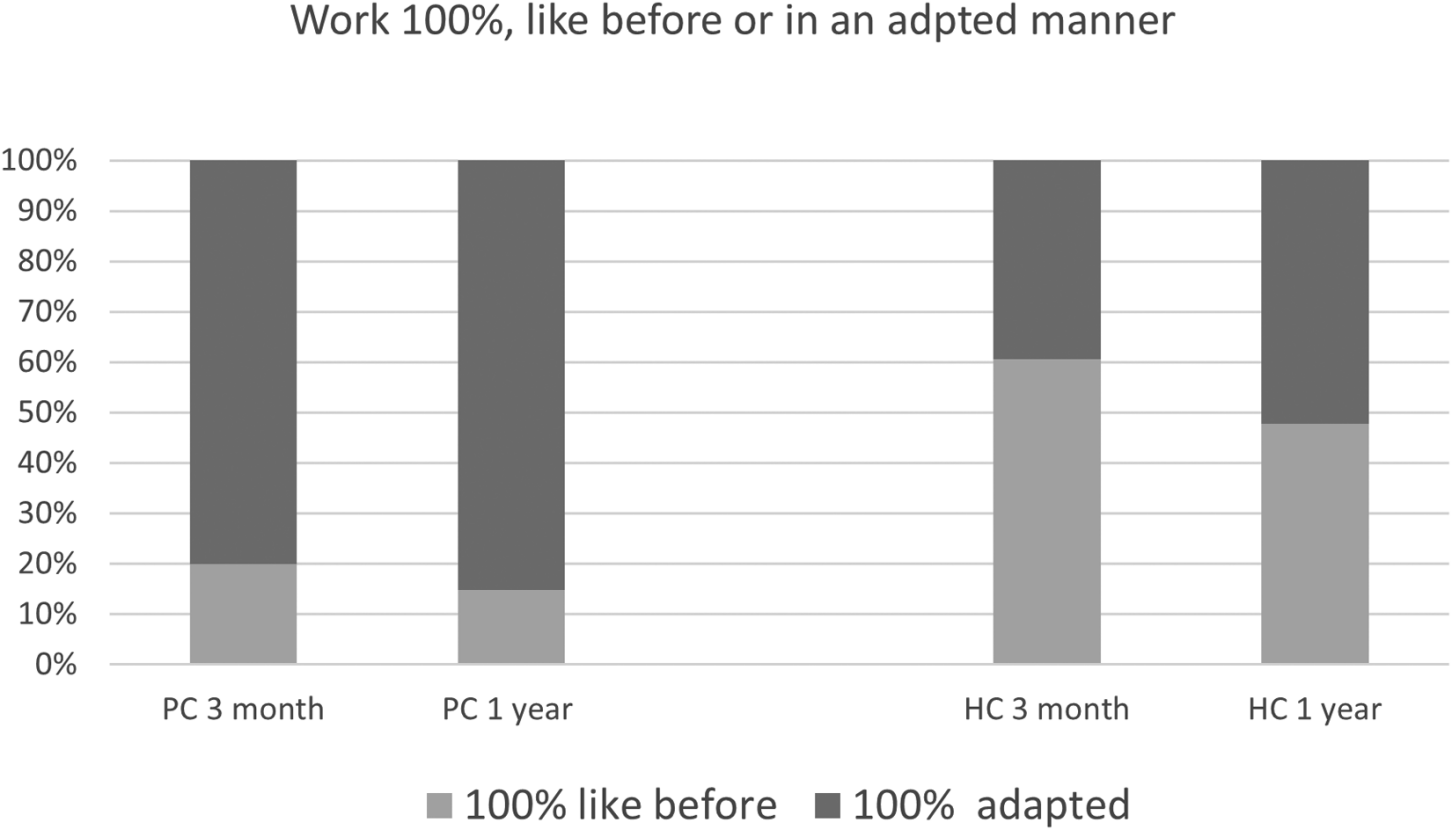
Percentages of participants in the PC and HC groups who worked 100%, either like before COVID or in an adapted manner, at 3 months and 1 year.

Some participants reported during their interviews that, although they had returned to full-time work, their performance was lower than before COVID-19 infection or they needed to adapt how they worked. This factor was explored by dividing the subjects into two groups: those who performed their work in a manner similar to that before COVID-19 and those who worked in an adapted manner (Figure 3). The percentage of subjects working in an adapted manner was high in both the HC and PC groups. At 12 months, the percentage working 100% in the same way as before COVID was significantly higher in the HC (46%) than in the PC (15%) group. All of those working full-time at 12 months in the PC+ group were doing so with adaptations. These adaptations included the option to work from home, as well as having an understanding manager who considered employees' needs in performing certain tasks, the need for more breaks and acceptance of slower performance. The participants also reported that they did not perform at the same level or as much as before COVID.

Taking as a reference that most of this cohort performs a theoretical job (*n* = 59/92 (64,5%)), it was observed that most of the people performing this type of work have returned to some work activity (*n* = 71(79,8%)), and almost half of the sample has returned to 100% (*n* = 49 (51%)), although also most of them have done so in an adapted form [*n* = 35 (33,7%)]. However, most people with post COVID-19 condition working in the service sector (*n* = 8 (8,6%) have not returned to their work activity one year after COVID- 19 infection. Regarding those doing physical work, the results are more variable, with [*n* = 5 (25%)] not having returned to work, [*n* = 7 (35%)] having returned 100% to work and [*n* = 4 (20%)] having returned to work in an adapted manner (Table 3).

**Figure 4:**
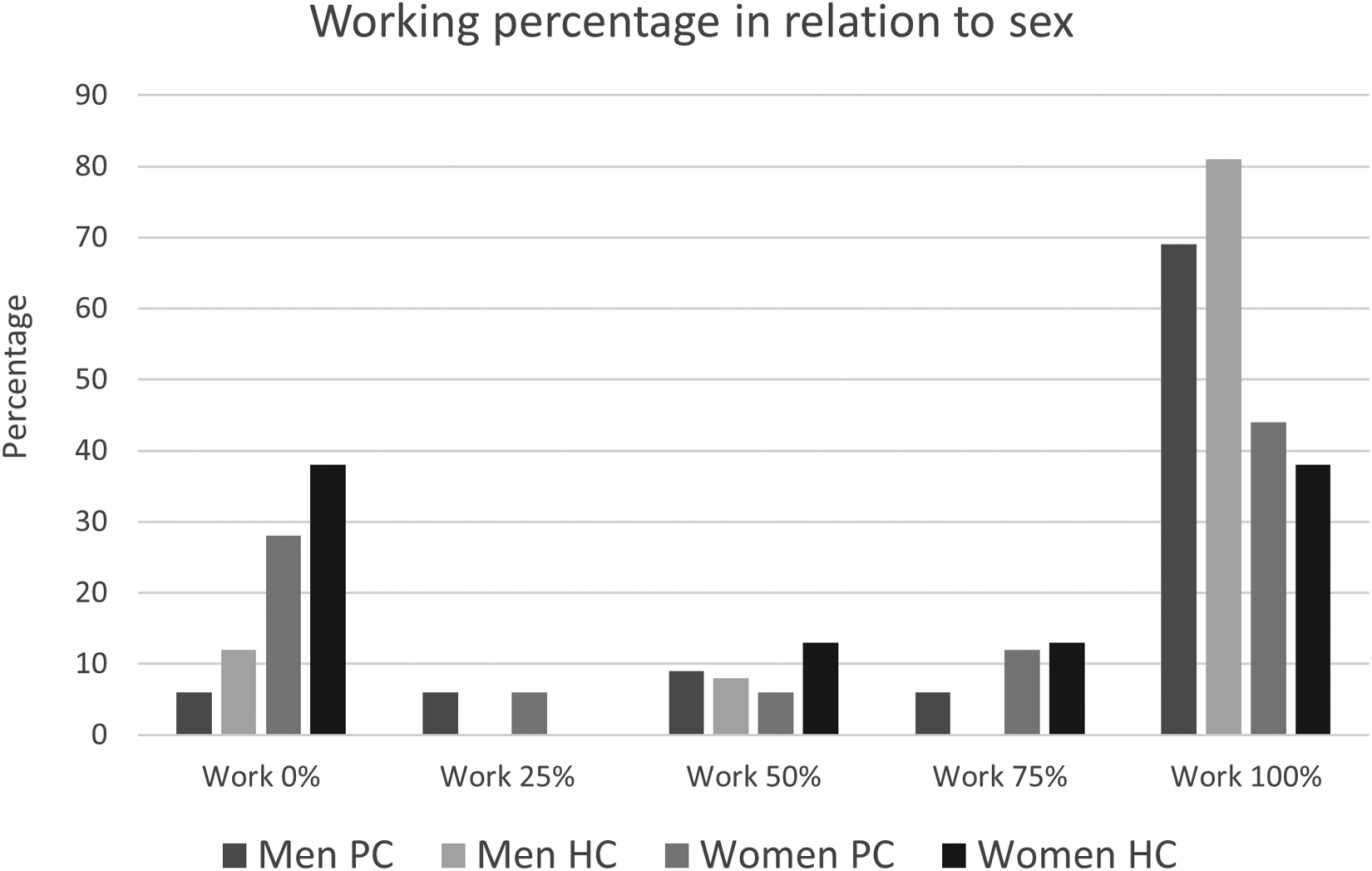
Employment rates of men and women in the HC and PC groups at one year.

To explore the RTW in relation to gender and age groups (±40 years) the different levels of return to work in the PC and HC groups from this perspective could be seen in Table 2. The HC group was older than the PC why the number of men in HC < 40 years was only 3 and therefore not valid to compare with the other groups in regard to RTW. In most of the groups in Table 2 there were high percentages that had returned to work 100% but with the need of adaptations at 1 year follow-up except for the women in the HC that tended to have less participants in adapted 100% work but more not in work at all compared to the PC group However, the percentage of women who do not RTW one year after being diagnosed with persistent covid is higher than that of men, regardless of whether they come from primary care (28.1%) or hospital (37.5%). The differences between men and women are significant, with women being 3.5 times more likely than men to remain unable to work one year after being diagnosed with persistent covid [Odds ratio 3.482 (CI 1.151–10.534); *z* = 2.209; *p* = 0.0272]. Women showed significantly lower proportions of working 100% as before and also the proportions not at all back in work were higher in women (ꭕ^2^ = 13.67 (*p* = 0.01). Being male makes it 12 times more likely to return to work as before the covid-19 pandemic than a female [Odds ratio 12.000 (CI 2.538–56.718); *z* = 3.136; *p* = 0.0017]. Of the women working full-time, only one, in the HC group, worked full-time without adjustments. By comparison, 15 men in both groups worked full-time without adjustments (Table 2 and Figure 4).

**Table 2:** Proportion with different levels of return to work split by gender and age in the two groups PC and HC.

1 year follow-up	0%	Part time	100% adapted	100% like before
PC women *n* = 34	28.1%	28.1%	43.8%	0%
HC women *n* = 16	37.5%	25.0%	25.0%	12.5%
PC men *n* = 16	12.5%	12.5%	50.0%	25.0%
HC men *n* = 26	11.5%	7.7%	42.3%	38.5%
PC < 40 years *n* = 17	29.4%	11.8%	52.9%	5.9%
HC < 40 years *n* = 3	66.7%	0%	0%	33.3%
PC > 40 years *n* = 31	19.4%	29.0%	41.9%	9.7%
HC > 40 years *n* = 31	12.9%	12.9%	32.3%	41.9%

**Table 3:** Description of different types of work and return to work (RTW) for the whole sample at 12-months follow-up after COVID-19 infection.

RTW	Unit	Whole sample	White collar work	Blue collar work	Service sector
		% (*n*)	% (*n*)	% (*n*)	% (*n*)
Total at 12 months	% (*n*)	88.2 (92)	64.5 (59)	26.9 (25)	8.6 (8)
No RTW	% (*n*)	20.2 (21)	14.3 (8)	25.0 (5)	62.5 (5)
100% as before	% (*n*)	15.4 (16)	14.3 (8)	35.0 (7)	0.0 (0)
100% adapted	% (*n*)	33.7 (35)	44.6 (25	20.0 (4)	25.0 (2)
Part time	% (*n*)	19.2 (20)	26.8 (15)	20.0 (4)	12.5 (1)

### Factors associated with RTW

Logistic regression analysis, of the whole sample, attempted to identify factors associated with RTW. The chosen model contained four independent variables (sex, age, group and type of work). The dependent variable (work or not work) was dichotomised to 0%–25% or >25%, respectively. The model containing all predictors was statistically significant [χ^2^ = 14.46, *p* < 0.01 (*n* = 82)], indicating that this model could distinguish RTW from non-RTW. Only one independent variable, sex, contributed significantly to this model indicating that a man was more than 3 times more likely to RTW than a woman [*p* = 0.03, odds ratio (OR) 3.74]; (Table 4). RTW rates were lower in women than in men, with 60% of women and 40% men not working or working ≤25%, regardless of group (Figure 4). The model explained between 16.2% (Cox and Snell R square) and 23.3% (Nagelkerke R Square) of the variance in RTW and correctly classified 71.7% of the participants. This model could therefore explain some of the conditions associated with RTW (Table 4).

**Table 4:** Logistic regression analysis of factors predicting return to work for the whole sample.

Variables (in the model)	B	S.E	Wald	df	Sig.	Exp (B)	95.0% C.I for odds ratio	
							Lower	Upper
Sex	1.32	0.67	4.59	1	0.032	3.74	1.12	12.5
Age	0.03	0.02	1.17	1	0.279	1.03	0.97	1.08
PC/HC (HC)	0.60	0.56	1.15	1	0.283	5.84	0.60	5.52
White collar work	−0.89	0.67	1.76	1	0.184	0.40	0.10	1.53
Blue collar work	−1.75	0.91	3.71	1	0.054	0.17	0.29	1.03
Constant	−0.92	1.25	0.54	1	0.461	0.39		

Logistic regression analysis was also performed to assess the impact of COVID-19 onset in different waves (I-III) on RTW. This analysis, using subjects diagnosed during wave one as a reference category, found that RTW differed significantly in subjects diagnosed during wave two, with the latter 5 times more likely to RTW (OR of 5.38). The likelihood of RTW did not differ significantly in subjects diagnosed during waves 1 and 3. This model explained between 10.9% (Cox and Snell R square) and 15.7% (Nagelkerke R Square) of the variance in RTW and correctly classified 71.7% of the study subjects (Table 5).

**Table 5:** Logistic regression analysis of the impact of wave of the pandemic on return to work.

Variables (in the model)	B	S.E	Wald	df	Sig.	Exp (B)	95.0% CI for odds ratio	
							Lower	Upper
Reference’ Wave 1			9.387	2	0.009			
Wave 2	1.683	0.569	8.753	1	0.003	5.383	1.765	16.419
Wave 3	1.153	0.848	1.847	1	0.174	3.167	0.601	16.692
Constant	0.234	0.307	0.579	1	0.447	1.263		

The impact of post-COVID symptoms on work ability as assessed by OTs based on interview descriptions at 12-months showed that in the PC group, 37% were rated as the post-COVID symptoms had a great impact on RTW and 28% were rated as post-COVID symptoms had a moderate impact on RTW. In the HC group, these percentages were 44% and 19%, respectively, and in the PC+ group, 78% were rated as post-COVID symptoms had a great impact hindering RTW. There was no significant difference in ratings between HC and PC group.

### Obstacles to working and underlying impairments and conditions after COVID

The result of the content analysis of the experiences of obstacles to work after the COVID generated seven categories; lack of energy, decreased physical capability to work, decreased mental stamina, reduced cognitive ability, increased sensitivity to stress, generally reduced capacity to carry out work tasks and lack of understanding and support (Table 6). These categories consist of sub-categories describing the impairments and conditions leading to barriers/obstacles in the work situation. The most evident obstacles mentioned by most respondents were a lack of energy and fatigue that limited working time, the tempo of performance and the type of work they could manage. Tasks were performed more slowly and with lower quality than before the COVID infection. Some of the participants experienced enormous fatigue even though they only worked part-time. In addition to adapting by reducing working hours, the participants also described how breaks between different tasks became necessary to cope with the energy shortage. A bus driver described;

**Table 6:** Categories and subcategories from the content analysis regarding obstacles to working and the consequences leading to barriers in the work situation.

Categories	Subcategories
Obstacles to working	Impairments and conditions leading to barriers in the work situation:
Lack of energy	• Limitation on the scope of work • Reduced tempo of performance • Lower quality in of activity performance • Longer time for recovery • More frequent rest
Decreased physical capability to work	• Difficulty climbing stairs and lifting heavy objects, • Gets out of breath easily • Orthostatic problems • Headache on exertion
Decreased mental stamina	• Lack of initiative, • Difficulty to spend time in stimuli-rich environment • The brain may “shut down”.
Reduced cognitive ability	• Decreased ability to concentrate • Difficulty in multitasking • Risk of forgetting information and agreements • Difficulty to cope with distractions • Difficulty to solve problems and draw conclusions
Increased sensitivity to stress	• Difficulty to understand and sort information • Difficulty to act appropriately • Decreased flexibility
Generally reduced capacity to carry out work tasks	• Need of support from others • Limited ability to perform certain tasks such as leadership • Difficulties to manage meetings with several people involved • Difficulty to fully hold in work that involves customer contact • Difficulty to take notes at meetings
Lack of understanding and support	• Insufficient support for adaptation • Requirements too high compared to level of ability

“I don't have the energy to do anything extra like asking passengers to show their ticket. I have stopped doing that. I don't have the energy to argue with people. When I get into an argument, it takes all the energy for the day.”

Participants reported that they needed much longer and more frequent recovery periods. In both the HC and PC groups the participants experienced lack of energy and decreased mental stamina, but it was more frequently mentioned in the PC group.

“I have adapted work tasks after the need of more frequent breaks. My attention is impaired, and I have difficulty with divided attention. This means a reduced stamina and need for breaks, which prevents me from working full-time.”

By contrast, most subjects in the HC group reported decreased physical capability to work that required physical efforts. Physical barriers to RTW included climbing stairs and lifting heavy objects. Decreased physical capability were frequently associated with respiratory and orthostatic problems and sometimes with pain. A nurse expressed it in the following way, and related her experiences to feelings of shame:

“My breathing is still affected, I get short of breath and the air is not enough, I gasp for breath. Embarrassing to be out of breath when I bend over. I am reluctant to go down the stairs to get things. I need more transition time between tasks.”

In combination with decreased mental stamina, participants reported difficulty to spend time in stimuli-rich environment, too many stimuli tire them or hinder cognitive processes. If the load is too high, then the brain may “shut down”. This decreased mental stamina creates a lack of initiative, and subsequently leads to the least possible effort being made. Participants also talked about the difficulty of managing the same workload as before.

A participant in a managerial position described the following:

“I feel more lacking in initiative and flatter in attitude. Before COVID I was also better at managing stress”.

Reduced cognitive ability was often reported. This experience was described in the category reduced cognitive ability and implied deficits in attention, working memory and speed of processing. Multitasking was difficult and the ability to concentrate was short-lived. Many participants experienced forgetfulness and were told that they needed reminders, which was embarrassing. Difficulties in coping with reduced cognitive abilities became more evident for participants with managerial positions as it meant difficulties in making decisions and remembering and retaining different cognitive processes and information in memory. A county council employee, described:

“I am hindered by having to read difficult texts, make decisions. I don't have the energy, and I find it hard to think about keeping all the parts together at the required pace when my energy is so limited. Embarrassing.”

Participants frequently had difficulties attending meetings with several other persons and were unable to take notes or draw conclusions. These participants often needed to rely on support from others. They reported being easily distracted and unable to sit in an activity-based office or the same room as many other persons. Generally reduced ability made leading positions too demanding and hindered the ability to converse with clients, which required flexibility and memory.

Many participants also found it more difficult to cope with stress and to understand and act in stressful situations, they showed an increased sensitivity to stress.

“I cannot work at all. I am far from being able to cope with mine tasks mainly due to lack of energy and both physical and cognitive dysfunktions. At the time of my illness, I worked as a social worker with family home placements. Now I am too impaired and have too much fatigue and weakness. Also difficult with cognitive flexibility, I can't cope physically either. Can't hold conversations or sort out what is said and compile it.”

In many cases the participants who returned to work to some degree had an understanding and supportive management, allowing different kinds of adaptations. By contrast, some of the subjects reported lack of understanding and supportive management, as obstacles for their RTW. Unrealistic demands and lack of understanding by supervisors made their jobs too burdensome. When the requirements were too high compared to their level of ability it could result in new sick leave.

A participant who has an office job in a car factory describes:

“It works with full time job because it has been possible to work from home. I would never have managed to go to work 5 days a week. It is very important that the boss is understanding, otherwise it would not have been possible. Just driving a car is an effort. I can't handle stress and pressure like before.”

## Discussion

The study showed that still after 12 months many subjects with post-COVID condition were unable to RTW full-time, without accommodations, because of persistent symptoms, such as fatigue and cognitive dysfunction. RTW did not differ significantly by occupation, but such probability was higher in men, especially if they had contracted COVID-19 infection in the second wave. No statistically significant differences were found between HC and PC group in the levels of RTW. However, looking at RTW for the different sexes showed that several times more women were unable to work at all and in need of adaptations to be able to work 100% at one year than men, and the proportion of women in the PC group were 2/3 compared to 1/3 in the HC.

The overall findings of our study are in line with other studies (22). Large studies have shown that 1%–2% of all patients infected with COVID-19 have long-term persistent symptoms (5, 23), with most cases of post-COVID condition being of working age (23). Although the highest overall cumulative incidence has been observed in patients with severe onset of the COVID-19 infection, most individuals diagnosed with post-COVID condition had not been hospitalized (5). Common persistent symptoms include fatigue, shortness of breath and cognitive dysfunction, which generally have a significant impact on everyday functioning. Symptoms may arise following initial recovery from an acute COVID-19 episode or persist from the initial illness and may also fluctuate or relapse over time (23). Long after onset of the COVID-19 infection, a substantial number of both hospitalised and non-hospitalised individuals have been unable to RTW (9, 24), a finding confirmed in the present study. Fatigue and cognitive dysfunction greatly affect the ability to work, with recovery from these symptoms being slow, as shown by minimal change between 3 and 12 months reported from the main study (8). Similarly slow increases in ability to work between 6 and 12 months have been reported, resulting in restricted work performance (9, 25, 26).

There are conflicting evidence regarding the association of acute severity and outcome. Some studies finds this association (13, 22) while others finds that RTW has not been associated with the initial severity of COVID-19 infection, demographic characteristics, lifestyle factors or performance on functional testing (9, 27). Another study of the patients assessed in the present study found that results on cognitive function tests were lower in the HC than in the PC group, whereas cognitive function had a greater impact on daily activities in the PC group. Persistent fatigue and cognitive dysfunction 1-year post-COVID had a great impact on daily activities (8). Moreover, a qualitative study reported that fatigue, cognitive dysfunction, respiratory symptoms and pain have a major impact on daily life and on professional or educational activities. Because these symptoms were unpredictable, cyclical and fluctuated over time, it was difficult to plan for RTW (28). Fatigue was found to be the strongest predictor of functional impairment after COVID-19, followed by depression and cognitive dysfunction (18). Interviews of subjects in the present study found that lack of energy, decreased mental stamina and reduced cognitive ability were major obstacles to functional ability and RTW.

Although initial COVID severity was not associated with RTW, other factors may explain some of the difficulties of RTW (27). The UK Military evaluated the ability of subjects with ongoing symptomatic or initially severe COVID-19 to return to full duty. Participants were categorised as fully deployable or medically downgraded, and factors associated with sustained long-term medical downgrading at 12 and 18 months were evaluated. Factors associated with medical downgrading 1 year after onset of COVID-19 infection included cognitive dysfunction, mental health symptoms, shortness of breath, fatigue and impaired cardiopulmonary function (in 51% of individuals). At 18 months, cognitive dysfunction, mental health and reduction in maximal aerobic capacity continued to be associated with inability to RTW in the 31% of individuals who remained occupationally restricted (27). A survey of over 3,000 subjects found cognitive dysfunction in 55% at 7 months, with 86% of working respondents experiencing reduced working ability. Combinations of neurological/cognitive and systemic symptoms showed the longest persistence (29). There is a heterogeneity in the reported proportions with persistent cognitive dysfunction that to some extent may be contributed to the instruments used and their sensitivity to detect dysfunction. As we point out in an earlier article (8) a reason for the conflicting results regarding cognitive function may be due to the use of crude screening instruments that do not capture more subtle impairments in a group of highly educated people with high demands on cognitive functioning in their work.

Although RTW 7 months after hospital discharge after COVID-19 infection may be common, these individuals may not have recovered completely, with work limiting participation in hobbies and physical activities due to fatigue and restricted participation (30). Due to persistent functional limitations after a COVID infection, adaptations are frequently required to allow people to resume work (17, 22, 28, 32–33). Interviews of subjects in the present study showed that many of those working full-time did so in an adapted way, such as working from home, performing only certain tasks, having more breaks, performing more slowly, and requiring flexibility in time and work content. The qualitative analyze also showed that some job roles were difficult to manage given the difficulties involved. This was the case, for example, for managerial roles and roles involving a lot of customer contact. Although RTW did not differ significantly in the PC and HC groups, the percentage requiring of adaptations was higher in the PC group. Logistic regression analysis found that group and type of work were unrelated to RTW, although blue collar work tended to have a negative impact on RTW. Both the percentage of subjects performing blue collar work and the existence of physical symptoms were higher in the HC group. One possible explanation for the result that type of work was not related to RTW could be the uneven distribution in the different work areas with very few of the participants working in the service sector and the majority having a white-collar work.

Logistic regression analyses in the present study also found that male sex and infection during the second wave of the pandemic were associated with a higher likelihood of RTW. This result could suggest a different pathogenicity in relation to its chronification between the different waves, a result that is supported, despite the small sample size of our study, by the results of the Center of Occupational and Environmental Medicine of Stockholm (COEMS). The COEMS found the risk of later RTW being twice as high in those infected during the first than during the second wave of the pandemic and 10% higher in women than in men (33). The present study found that, compared with onset during the first wave, onset during the third wave did not affect RTW, which may have been due to the low number of patients in the study infected during the third wave and the large 95% confidence interval in this group. The percentages of men and participants with onset during the second wave were significantly higher in the HC than in PC group, suggesting that male sex and onset during the second wave may be predictors of RTW mainly in the HC group. RTW, however, did not differ significantly in these two groups, although the percentage of HC, but not PC, subjects who attained RTW increased significantly from 3 to 12 months. In addition, the proportion of subjects fully able to work was higher in the HC group, while the proportion of subjects fully able to work with adaptations only was higher in the PC group. This could suggest that the HC with predominantly men with respiratory problems at 3 months still were highly affected but that the recovery from this was relatively good. Possibly this can also explain the fact that the PC group described significantly more barriers to daily activity due to cognitive dysfunction than HC and the cognitive dysfunctions have been shown to be one of the most persistent limitations (9).

Similar to the present study, the COEMS reported that type of work, demands at work and duration of sick leave were unrelated to RTW, although individuals in an administrative profession tended to have more sick days (34). Most of the subjects in the present study performed white collar work, with many of those working full-time requiring adaptations, indicating remaining difficulties. The ability to make adaptations may differ by types of work, making it more difficult to compare RTW in subjects performing different types of work.

The RTW process may be difficult, with many patients experiencing several relapses and several attempts to RTW (17). The slow and lengthy process of RTW may be due to the episodic nature of the condition (31) and the persistence of symptoms arising after attempts to work. The main obstacles to RTW include interactions between symptoms and jobs, lack of control over job pressures, inappropriate management policies regarding absence for sickness, and lack of COVID-aware cultures (17). RTW may be facilitated by communications, support and work adjustments (22, 32, 34). Adaptations can include work-from-home accommodations, allowing employed individuals to conserve energy, elevate their legs and pace in ways not feasible when physically present in an office. Understanding by management and colleagues (31), self-management support, modified work and graded return planning are important in promoting RTW (17). The recognition that long COVID is an occupational disease, following acute COVID-19, would provide better social protection (28) and support RTW.

As described in a recent meta-analysis (35), long haulers following viral illnesses are not specific to COVID-19. Symptoms like PCC have been described following past influenza pandemics since the late 19th century and similarities between PCC and myalgic encephalomyelitis/chronic fatigue syndrome have been well described. However, although similarities to other syndromes the PCC includes specific types of sequelae that are less common in other viral diseases (14). There are studies on post-viral and post-COVID-19 syndrome trials regarding treatment but more knowledge on how to design treatment and rehabilitation services are needed. There is also a need for more detailed and longer follow-up of cognitive dysfunction and fatigue and its impact on the ability to resume everyday activities and return to work. In addition, the literature show that women have the highest prevalence of long-term sick-leave among the working population in Sweden (36, 37) and disability pension has also been found to be greater for female workers in Sweden and other Scandinavian countries (38). People with recurrent COVID-19 sick leave were found to be significantly older and more often women than people with shorter period(s) of sick leave (<35 days) (39) why it will be important with further research on gender differences.

### Limitations and strengths

One limitation of this study was that the two groups were not completely comparable, as they differed in assessment times and circumstances for inclusion. The PC group was recruited from a selected group of patients who had consulted a primary care facility due to persisting problems after COVID-19 infection, whereas the HC group consisted of all patients with COVID-19 infection at the hospital. The results, however, were not affected by time of inclusion, as results were similar in the PC and HC group, and not in favor of PC despite the longer time for recovery. As sex showed differences in RTW it could be seen as a limitation that the groups differed in the proportions of men and women. However, as this was an explorative study this could also be seen as a strength that there were indications of sex differences in how they were affected. The comparisons provided insight into differences in symptoms and obstacles in patients with severe and milder acute illness, which in both groups persisted and limited RTW. Information regarding rehabilitation provision during the year after onset was not collected which also is a limitation. However, rehabilitation interventions other than hospitalization are offered in primary care at the request of the patient and thus provided the same conditions in both groups. Another limitation was the small numbers of participants infected during the third wave of the pandemic and in service jobs, which may have affected the association of RTW with pandemic wave and type of work. Logistic regression analysis, showed differences in RTW between the first and second waves, in agreement with previous findings (33).

The proportion of subjects not working at 12 months was highest in those performing service jobs, suggesting that the inclusion of more participants in service jobs may have resulted in an association between service jobs and RTW. The inclusion criteria regarding the Swedish language may have influenced the number in service jobs, as many of the patients admitted to hospital early in the pandemic were bus and taxi drivers, occupations common among immigrants, who may have been excluded due to language difficulties. Physical work tended to be a negative predictor of RTW, with the highest percentage of subjects in the HC group performing physical work. For example, another study found greater limitations for work and everyday life in COVID patients experiencing cardiopulmonary symptoms (40). Evaluation of a larger sample especially from the HC group may have shown different results, as the percentages reporting respiratory problems and physical limitations were higher in the HC than in the PC group. Overall, the study would have benefited from a larger sample that maybe could have detected more aspects explaining RTW. Since the participants in the present study were included from a main study with an older population, especially in the HC, the number of participants of working age were limited.

A more detailed grouping of occupations may also have provided greater insights that could help in vocational rehabilitation. Finally, the interviews focused on activity and the main restrictions in daily activities, with most questions addressing the most prominent symptoms, such as fatigue and cognitive symptoms. The results of this study may have been enhanced by detailed questions about oxygenation and autonomic problems, which are major limitations for many subjects and could affect RTW. Nevertheless, one strength of this study was the qualitative input from the interviews that could help explain the low rates of RTW. This study also provides understanding of the need for long-term rehabilitation and support for RTW after a COVID-19 infection.

## Conclusion

The study showed that RTW may be limited after COVID-19 infection, regardless of its initial severity. Gender differences were present and shown in the study with women being 3.5 times more likely than men to remain unable to work one year after being diagnosed with persistent covid. The obstacles for RTW were persistent symptoms such as fatigue, cognitive dysfunction and breathlessness with consequences as lack of energy, decreased physical capability, decreased mental stamina, reduced cognitive ability, increased sensitivity to stress and general reduced capacity. However, the support from employers and the environment also has an impact on the RTW. If necessary, appropriate rehabilitation measures should be offered and adaptations of work content and the organization of the work at the workplace are of great importance. Future research should focus on finding explanations for why women are so highly affected by PCC. Furthermore, in-depth knowledge should be developed about which cognitive functions are particularly vulnerable when falling ill with COVID-19 in order to be able to offer appropriate strategies that can improve the conditions for returning to work.

## Data Availability

The raw data supporting the conclusions of this article will be made available by the authors, without undue reservation.
