# Supplementary material for: Persistent obstacles for return to work after COVID-19 infection – an explorative follow-up study in Sweden
Source: Front Rehabil Sci. 2025 Sep 18;6:1628490. doi: 10.3389/fresc.2025.1628490 (PMC12490236; doi:10.3389/fresc.2025.1628490)
Supplement: Supplementary file 1 [file Datasheet1.pdf]

COVID-19 Follow-up

Date________________ **Personal Number**________________________________

Name____

Follow-up____________

Which symptoms persist at this follow-up?

Fatigue Sleepiness (need to sleep long or during the day)

Yes ☐ No ☐

Expand____
____
____
____
____

Weakness (time for activity, ability to walk, muscle weakness)

Yes ☐ No ☐

Expand____
____
____
____
____

Mental fatigue

Yes ☐ No ☐

Expand____
____
____
____
____

Cognitive impact (concentration, memory, etc.)

Yes ☐ No ☐

Expand____
____
____

How does fatigue affect you? To what extent do you need to rest? How is your sleep?

Expand____

How much fatigue is experienced today in general? Mark with a cross

Not at all ☐ ☐ ☐ ☐ ☐ ☐ ☐ ☐ ☐ ☐ Extremely much

Tremors in hands, impact on hand function

Yes ☐ No ☐

Breathing (shortness of breath, increased pulse, etc.)

Yes ☐ No ☐

Expand how it affects____

Other

Yes ☐ No ☐

Expand____

Personal care? (Difficulties, stamina, aids). Compare to before COVID

Expand____

____

____

____

____

How do daily chores work? Household, cleaning, laundry, gardening, repairs, shopping. Compare to before COVID

Expand____

____

____

____

____

What leisure activities do you have and how do they work? Interests, social contexts, physical activity. Compare to before COVID

Compare to before COVID

Expand____

____

____

____

____

Questions about WORK

Did you work before falling ill?

Yes ☐ No ☐

To what extent are you back at work?

Not at all ☐ 25% ☐ 50% ☐ 75% ☐ 100% ☐

When did you start working after falling ill and when were the different steps in returning to work?

-

____

____

____

Where do you work and what were the tasks before and now?

Compare to before COVID

Expand____

____

____

____

____

Are there impairments that affect your ability to work? In what way?

Compare to before COVID

Expand____

____

____

____

____

To what extent has the illness affected your mental health?

Compare to before COVID

Expand____

____

____

____

____
